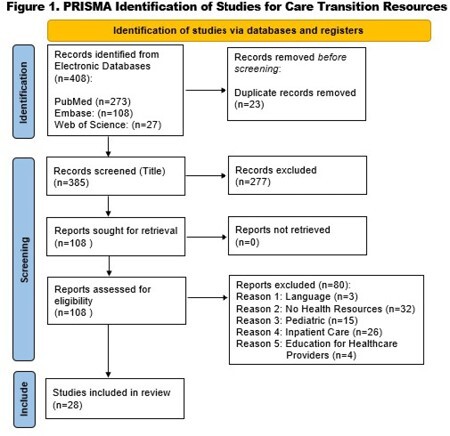# 728 REsource Support to Optimize REcovery (RESTORE) Study Scoping Review: Care Transition Resources for Burn Survivors

**DOI:** 10.1093/jbcr/irae036.271

**Published:** 2024-04-17

**Authors:** Mariana Velasquez-Cano, Camille Carnevale, Lauren Shepler, Diana L Tenney, Lewis E Kazis, Colleen M Ryan, Jeffrey C Schneider, Mary D Slavin

**Affiliations:** Department of Physical Medicine and Rehabilitation, Harvard Medical School, Spaulding Rehabilitation Hospital, Charlestown, Massachusetts, Medellin, Antioquia; University of Maryland, Boston, MA; Spaulding Rehabilitation Hospital, Harvard Medical School, Boston, MA; BH-BIMS, New Bedford, MA; Department of Health Law, Policy and Management, Boston University School of Public Health, Boston, MA; Massachusetts General Hospital/Shriners Children's, Boston, MA; Department of Physical Medicine and Rehabilitation, Harvard Medical School, Spaulding Rehabilitation Hospital, Charlestown, Massachusetts, Medellin, Antioquia; University of Maryland, Boston, MA; Spaulding Rehabilitation Hospital, Harvard Medical School, Boston, MA; BH-BIMS, New Bedford, MA; Department of Health Law, Policy and Management, Boston University School of Public Health, Boston, MA; Massachusetts General Hospital/Shriners Children's, Boston, MA; Department of Physical Medicine and Rehabilitation, Harvard Medical School, Spaulding Rehabilitation Hospital, Charlestown, Massachusetts, Medellin, Antioquia; University of Maryland, Boston, MA; Spaulding Rehabilitation Hospital, Harvard Medical School, Boston, MA; BH-BIMS, New Bedford, MA; Department of Health Law, Policy and Management, Boston University School of Public Health, Boston, MA; Massachusetts General Hospital/Shriners Children's, Boston, MA; Department of Physical Medicine and Rehabilitation, Harvard Medical School, Spaulding Rehabilitation Hospital, Charlestown, Massachusetts, Medellin, Antioquia; University of Maryland, Boston, MA; Spaulding Rehabilitation Hospital, Harvard Medical School, Boston, MA; BH-BIMS, New Bedford, MA; Department of Health Law, Policy and Management, Boston University School of Public Health, Boston, MA; Massachusetts General Hospital/Shriners Children's, Boston, MA; Department of Physical Medicine and Rehabilitation, Harvard Medical School, Spaulding Rehabilitation Hospital, Charlestown, Massachusetts, Medellin, Antioquia; University of Maryland, Boston, MA; Spaulding Rehabilitation Hospital, Harvard Medical School, Boston, MA; BH-BIMS, New Bedford, MA; Department of Health Law, Policy and Management, Boston University School of Public Health, Boston, MA; Massachusetts General Hospital/Shriners Children's, Boston, MA; Department of Physical Medicine and Rehabilitation, Harvard Medical School, Spaulding Rehabilitation Hospital, Charlestown, Massachusetts, Medellin, Antioquia; University of Maryland, Boston, MA; Spaulding Rehabilitation Hospital, Harvard Medical School, Boston, MA; BH-BIMS, New Bedford, MA; Department of Health Law, Policy and Management, Boston University School of Public Health, Boston, MA; Massachusetts General Hospital/Shriners Children's, Boston, MA; Department of Physical Medicine and Rehabilitation, Harvard Medical School, Spaulding Rehabilitation Hospital, Charlestown, Massachusetts, Medellin, Antioquia; University of Maryland, Boston, MA; Spaulding Rehabilitation Hospital, Harvard Medical School, Boston, MA; BH-BIMS, New Bedford, MA; Department of Health Law, Policy and Management, Boston University School of Public Health, Boston, MA; Massachusetts General Hospital/Shriners Children's, Boston, MA; Department of Physical Medicine and Rehabilitation, Harvard Medical School, Spaulding Rehabilitation Hospital, Charlestown, Massachusetts, Medellin, Antioquia; University of Maryland, Boston, MA; Spaulding Rehabilitation Hospital, Harvard Medical School, Boston, MA; BH-BIMS, New Bedford, MA; Department of Health Law, Policy and Management, Boston University School of Public Health, Boston, MA; Massachusetts General Hospital/Shriners Children's, Boston, MA

## Abstract

**Introduction:**

Burn survivors report limited available resources to assist in their transition to the community after hospital discharge. This literature scoping review is designed to increase our understanding of the current burn aftercare landscape. Aims were to identify resources as well as assess barriers and facilitators available to burn survivors and their families aftercare.

**Methods:**

The Preferred Reporting Items for Systematic reviews and Meta-Analyses (PRISMA) served as a guideline to examine the following databases: PubMed, EMBASE and Web of Science. The search terms used included: burn(s), health resources, aftercare, after treatment, follow-up care, patient discharge, delivery of health care, ambulatory care, mental health and functional status. Criteria for inclusion were: burn injury diagnosis, outpatient or inpatient resources related to post-discharge burn recovery, location of resource delivery (burn center, outpatient clinic or other hospital setting), adult population, publications from 2012 to 2023 and in the English language. The International Classification of Functioning, Disability and Health (ICF) provided a conceptual framework to organize findings.

**Results:**

Of the 385 articles that were screened, 27 met inclusion criteria (Figure 1). Studies reported health resources within the following ICF Component (domains): Body Function (emotional function), Activities and Participation (work and employment, mobility), Environmental Factors (family and societal attitudes, social security and health services, education, general products related to technology such as telehealth. Implementation strategies included early planning and coordination of a multidisciplinary burn team to connect family and burn survivors with available resources. Educational materials to engage patients with outpatient care were also recommended. Examples of successful resources and services include: work accommodation, telehealth, peer support, web-based community online chat rooms and aftercare reintegration programs.

**Conclusions:**

This review provided an overview of current practices used to support burn survivors following discharge. Classification of these resources using the ICF framework fosters an understanding of the bio-psycho-social needs of the population and outlines an approach to design comprehensive aftercare services. This literature review is the initial step to develop a map of the resources currently offered to burn survivors and their families. Future qualitative work will build upon these findings and expand our understanding of the barriers and facilitators to meet the long term needs of people living with burn injury.

**Applicability of Research to Practice:**

This review highlights the breadth of post discharge resources currently described in the literature, providing a foundation for future long-term care recommendations.